# Anti-PLA2R antibodies: implications for therapeutic planning and the prediction of remission in membranous nephropathy

**DOI:** 10.1590/2175-8239-JBN-2026-E009en

**Published:** 2026-04-27

**Authors:** Gianna Mastroianni-Kirsztajn, Michelle T. Passos Riguetti, Juliana Mansur Siliano

**Affiliations:** 1 Universidade Federal de São Paulo, Departamento de Medicina, Disciplina de Nefrologia, São Paulo, SP, Brazil.

The availability of serum antiphospholipase A2 receptor antibody (anti-PLA2R) levels in nephrology is a laboratory resource with clinical applicability for the diagnosis and management of glomerular diseases, particularly primary membranous nephropathy^1^.

In the current edition of the BJN, the article “Anti-PLA2R antibody reduction as a predictor of treatment response in primary membranous nephropathy” by Bashir et al.^2^ was published, which discusses the use of this test.

The authors aimed to evaluate the levels of these autoantibodies in patients with primary membranous nephropathy (pMN) undergoing immunosuppressive treatment, who had these measurements taken at baseline and 6 months after treatment initiation. They observed that patients with lower baseline levels of anti-PLA2R (and higher serum albumin), as well as a reduction in antibody levels 6 months after starting one of the immunosuppressive treatments, more frequently experienced disease remission. It is worth noting that, just as there was a reduction in anti-PLA2R autoantibody levels, proteinuria levels decreased and serum albumin levels increased after 6 months of treatment. However, the authors comment, as an advantage of anti-PLA2R testing, that the decrease in antibody levels preceded the changes in the levels of the other tests. Finally, they concluded that performing anti-PLA2R assays at 6-month intervals is a useful tool for decision-making in the management of pMN^2^.

Although several publications on the subject have already been carried out, Bashir et al.^2^ highlighted that most of them involved Western populations. Therefore, they considered it necessary to evaluate other populations, including the Pakistani population, which was included in their current study. This concern has indeed motivated numerous researches with different approaches around the world^3,4^, given the possibility that genetic factors, among others, could interfere with the profile and the applicability of this test in different regions; however, in general, it has been used successfully worldwide. For example, in a study involving 117 Brazilian patients with glomerulopathies, it was demonstrated that positivity for anti-PLA2R had high specificity and positive predictive value for the diagnosis of pMN and a strong correlation with disease activity. It also became clear in that study that the best situation for a single-use test would be to perform the assay in patients with active disease before the start of immunosuppressive treatment^3^.

The relevance of anti-PLA2R measurement in MN stems mainly from the well-established participation of these autoantibodies in the pathogenesis of most cases of pMN, while other antibodies identified subsequently contribute to the development of a smaller proportion of primary forms and of secondary forms^1^.

From several studies with this biomarker in pMN, a diversity of roles for anti-PLA2R antibodies has been described, among which the following can be cited: the confirmatory diagnosis of the primary form of MN; possible association of absolute autoantibody levels with prognosis and response to treatment; association of their levels with the immunological activity of the disease; association of decreased anti-PLA2R levels with a higher probability of response; and the diagnosis of post-transplant MN^1,3^. In addition, Bashir et al.^2^ state that this biomarker is important in clinical practice for personalized management and treatment adjustment.

The contribution of anti-PLA2R testing to clinical practice is undeniable, but we believe that one of the points requiring better definition is the recommended interval for repeating the test. It is known that some centers choose to measure anti-PLA2R in the third month after the start of immunosuppression and make decisions regarding the current treatment at that time. Although international guidelines recommend repeating anti-PLA2R testing in 3–6 months^5^, with the shorter interval adopted in cases of high baseline levels, data from the article itself showed that, at 6 months, more readily available tests, such as serum albumin and proteinuria, also indicated the probability of remission or remission itself. Certainly, repeating anti-PLA2R testing adds information, but we consider that the best time to perform it should be justified by its impact on management, also considering the cost–benefit ratio on an individual basis, especially in regions with fewer resources allocated to healthcare.

Regarding this issue, Rojas-Rivera et al.^6^ suggest that timely clinical decisions can be made as early as 3 months of immunosuppression, making it possible to predict clinical remission of nephrotic syndrome within 2 years. They demonstrated that, at this point, the combined assessment of tests (24-hour proteinuria, serum creatinine, and immune response with anti-PLA2R) performed better than each of these parameters individually.

Another aspect that requires attention is the cut-off points for predicting remission described by Bashir et al.^2^, which cannot be generalized due to limitations inherent to their study, such as the retrospective design and the inclusion of a small number of patients from a single region, among other reasons.

Finally, Bashir et al.^2^ concluded that lower initial levels of anti-PLA2R or a reduction in their levels after the initiation of immunosuppression were associated with remission of pMN, corroborating previous findings. It is worth highlighting, in addition to these, among the various roles already described for anti-PLA2R, that high or rising levels are associated with nephrotic syndrome and progressive loss of renal function, supporting the initiation of immunosuppressive therapy; the reappearance or increase in the titers of these antibodies precedes relapse; and the persistence or reappearance of anti-PLA2R antibodies after kidney transplantation predicts the development of recurrent MN. Taken together, these clinical applications of anti-PLA2R establish it as an unquestionable biomarker in the investigation and monitoring of glomerulopathies.

## Data Availability

No new data were generated or analyzed in this study.
