# Supplementary material for: Anti-PLA2R antibodies: implications for therapeutic planning and the prediction of remission in membranous nephropathy
Source: J Bras Nefrol. 2026 Apr 27;48(2):e2026E009. doi: 10.1590/2175-8239-JBN-2026-E009en (PMC13120714; doi:10.1590/2175-8239-JBN-2026-E009en)
Supplement: Translation

# Translation: Anticorpos anti-PLA2R: implicações na programação terapêutica e previsão de remissão na nefropatia membranosa

**DOI:** 10.1590/2175-8239-JBN-2026-E009pt

**Authors:** Gianna Mastroianni-Kirsztajn, Michelle T. Passos Riguetti, Juliana Mansur Siliano

**Affiliations:** 1 Universidade Federal de São Paulo, Departamento de Medicina, Disciplina de Nefrologia, São Paulo, SP, Brasil.

A disponibilidade da dosagem sérica de anticorpos anti-receptor de fosfolipase A2 (anti-PLA2R) introduziu na nefrologia um recurso laboratorial com aplicabilidade clínica para o diagnóstico e manejo de doenças glomerulares e, em especial, da nefropatia membranosa primária (NMp)^1^.

Na edição atual do BJN, foi publicado o artigo “Redução dos níveis do anticorpo anti-PLA2R como preditor de resposta ao tratamento na nefropatia membranosa primária” de Bashir et al.^2^, que trata da utilização do referido teste.

Os autores se propuseram a avaliar os níveis desses autoanticorpos em pacientes com NMp submetidos a tratamento, os quais realizaram tais dosagens no período basal e aos 6 meses após o início da imunossupressão. Observou-se que pacientes com níveis basais mais baixos de anti-PLA2R (e níveis mais elevados de albumina sérica), bem como aqueles que apresentaram redução desses anticorpos após 6 meses do início de um dos imunossupressores, evoluíram mais frequentemente com remissão. Vale salientar que, assim como houve redução dos níveis de anti-PLA2R, os níveis de proteinúria caíram e os de albumina sérica se elevaram após 6 meses de tratamento. No entanto, como vantagem da dosagem de anti-PLA2R, os autores destacam que a redução dos níveis de autoanticorpos antecedeu as alterações observadas nos demais exames. Por fim, concluíram que a realização das dosagens desses autoanticorpos com intervalo de 6 meses constitui um recurso útil para a tomada de decisão no manejo da NMp^2^.

Ainda que diversas publicações sobre o tema já tenham sido realizadas, eles ressaltaram que a maioria delas envolveu populações ocidentais, de modo que consideravam necessária a avaliação de outras populações, entre as quais a paquistanesa, que foi contemplada no seu atual estudo^2^. Essa preocupação motivou, de fato, diversos trabalhos, com diferentes abordagens, em todo o mundo^3,4^, diante da possibilidade de que fatores genéticos, entre outros, interferissem no perfil e na aplicabilidade desse teste em diferentes regiões. No entanto, de um modo geral, a ferramenta tem sido utilizada com sucesso mundialmente. Em estudo envolvendo 117 pacientes brasileiros com glomerulopatias, por exemplo, demonstrou-se que a positividade para anti-PLA2R teve especificidade e valor preditivo positivo elevados para o diagnóstico de NMp e forte correlação com a atividade. Ficou evidente também que a melhor situação para uso único do teste seria fazer a dosagem com doença ativa, antes do início da imunossupressão^3^.

A relevância da dosagem de anti-PLA2R em NM decorre, sobretudo, da participação bem estabelecida desses autoanticorpos na patogênese da maior parte dos casos de NMp, enquanto outros anticorpos, posteriormente identificados, contribuem para o desenvolvimento de uma proporção menor de formas primárias e, também, para as secundárias^1^.

A partir de vários estudos com esse biomarcador em NMp, descreveu-se uma diversidade de papéis para os anticorpos anti-PLA2R, entre os quais podem ser citados: o próprio diagnóstico confirmatório da forma primária da NM; possível associação dos níveis absolutos dos autoanticorpos com prognóstico e resposta ao tratamento; associação dos seus níveis com a atividade imunológica da doença; associação da redução dos níveis de anti-PLA2R com maior probabilidade de resposta; diagnóstico de NM pós-transplante^1,3^. Além disso, Bashir et al.^2^ enfatizam que esse biomarcador é importante na prática clínica para manejo personalizado e ajuste de tratamento.

É inegável a contribuição da dosagem de anti-PLA2R na prática clínica, mas consideramos que, entre os pontos que exigem uma melhor definição, está o intervalo recomendado para a repetição do teste. É sabido que alguns centros optam por dosar anti-PLA2R no terceiro mês após o início da imunossupressão, tomando decisões sobre o tratamento vigente nesse momento. Ainda que diretrizes internacionais recomendem repetição da dosagem de anti-PLA2R em 3 a 6 meses^5^, adotando-se o intervalo menor em caso de níveis basais elevados, foi possível observar, com dados do próprio artigo aqui discutido, que, aos 6 meses, exames disponíveis de forma mais rápida e mais fácil, como a albumina sérica e a proteinúria, também já indicavam a probabilidade de remissão ou a remissão propriamente dita. Certamente, a repetição da dosagem de anti-PLA2R acrescenta informação; porém, consideramos que o melhor momento para realizá-la deve ser justificado por seu impacto na conduta, ponderando também o custo-benefício de forma individualizada, sobretudo em regiões com menos recursos destinados à saúde.

Em relação a essa questão, Rojas-Rivera et al.^6^ sugerem que decisões clínicas oportunas já podem ser tomadas após 3 meses de imunossupressão, possibilitando prever a remissão. Eles demonstraram que, nesse momento, a avaliação combinada de testes (proteinúria de 24 horas, creatinina sérica e resposta imunológica com anti-PLA2R) teve melhor desempenho do que cada um desses parâmetros individualmente.

Outro aspecto que exige atenção são os pontos de corte para previsão de remissão descritos por Bashir et al.^2^, que não podem ser generalizados, devido às limitações inerentes ao estudo, como o desenho retrospectivo e a inclusão de um número pequeno de pacientes, provenientes de uma só região, entre outros motivos.

Por fim, Bashir et al. concluíram que níveis iniciais mais baixos de anti-PLA2R ou redução dos seus níveis após a instituição de imunossupressão associaram-se à remissão da NMp, o que confirma estudos anteriores. Vale destacar, além desses, entre os papéis já descritos para o anti-PLA2R, que níveis altos ou em elevação estão associados à síndrome nefrótica e à perda progressiva da função renal; o reaparecimento ou aumento dos seus títulos precede a recidiva; e a persistência ou o reaparecimento de anti-PLA2R após o transplante renal prediz o desenvolvimento de NM recorrente. Sem dúvida, essas formas de aplicar, na prática clínica, o anti-PLA2R fazem dele um biomarcador com posição inquestionável na investigação e no monitoramento da NMp.

## Data Availability

Nenhum dado novo foi gerado ou analisado neste estudo.
